# A cross-sectional examination of school characteristics associated with overweight and obesity among grade 1 to 4 students

**DOI:** 10.1186/1471-2458-13-982

**Published:** 2013-10-20

**Authors:** Scott T Leatherdale

**Affiliations:** 1 School of Public Health and Health Systems, University of Waterloo, 2000 University Avenue, Waterloo, ON N2L 3G1, Canada

**Keywords:** Obesity, Body mass index/BMI, Built environment, Physical activity, Children

## Abstract

**Background:**

Excessive weight gain among youth is an ongoing public health concern. Despite evidence linking both policies and the built environment to adolescent and adult overweight, the association between health policies or the built environment and overweight are often overlooked in research with children. The purpose of this study was to examine if school-based physical activity policies and the built environment surrounding a school are associated with weight status among children.

**Methods:**

Objectively measured height and weight data were available for 2,331 grade 1 to 4 students (aged 6 to 9 years) attending 30 elementary schools in Ontario, Canada. Student-level data were collected using parent reports and the PLAY-On questionnaire administered to students by study nurses. School-level policy data were collected from school administrators using the Physical Activity Module of the Healthy School Planner tool, and built environment data were provided by the Enhanced Points of Interest data resource. Multi-level logistic regression models were used to examine the school- and student-level characteristics associated with the odds of a student being overweight or obese.

**Results:**

There was significant between-school random variation in the odds of a student being overweight [σ^2^_μ0_ = 0.274(0.106), p < 0.001], but not for being obese [σ^2^_μ0_ = 0.115(0.089)]. If a student attended a school that provided student access to a variety of facilities on and off school grounds during school hours or supported active transportation to and from school, he/she was less likely to overweight than a similar student attending a school without these policies. Characteristics of the built environment were not associated with overweight or obesity among this large cross-sectional sample of children.

**Conclusions:**

This new evidence suggests that it may be wise to target obesity prevention efforts to schools that do not provide student access to recreation facilities during school hours or schools that do not support active transportation for students. Future research should evaluate if school-based overweight and obesity prevention programming might be improved if interventions selectively targeted the school characteristics that are putting students at the greatest risk.

## Background

Excessive weight gain among youth is an ongoing public health concern. Data from the 2007–09 Canadian Health Measures Survey suggest that among Canadian youth aged 6 to 10, 35.6% of boys and 17.5% of girls are overweight [1]. Considering that the prevalence of obesity has increased dramatically in the past two decades [1], it appears that modifiable factors (e.g., declines in physical activity (PA)) are likely more important determinants of the current crisis than non-modifiable factors (e.g., genetics).

Research has previously identified that overweight youth are less likely to be active [2-4], more likely to spend time in sedentary behaviours [2,3,5], and more likely to be male [2]. Although research has identified that dietary factors are associated with overweight among adolescents, data from the Continuing Survey of Food Intake for Individuals (CSFII) and the National Health and Nutrition Examination Survey III (NHANES) [4], and a recent research review [6], suggest that dietary factors are not strongly associated with overweight in children. It would be informative to also understand how the different preferences youth have for being active are associated with their weight status as affective attitudes (i.e., preferences for behaviour) are an important predictor of children’s PA [7] and because student preferences are amenable to modification.

Ecological models suggest that weight status would not just be determined by individual characteristics alone, but also by the characteristics of the environmental context (e.g., school) in which that individual is situated [8]. Research among older youth has identified the school environment as being an important context for weight status [9-17]. For instance, existing evidence suggests that there is modest variability in overweight between schools (~2-5%), demographic characteristics (e.g., school size and school socioeconomic status) appear to be associated with the risk of overweight [17], and school-based PA programs do not appear to improve body mass index (BMI) in children [18]. We also know that there is variability in student PA levels across schools and that different school characteristics provide youth with different opportunities to engage in PA [11,13], suggesting it is important to examine youth activity when exploring school variability in overweight. Given the lack of evidence exploring (a) the importance of the school environment on weight status among younger children, and (b) how PA *policies* within the school environment are associated with weight status, there remains an important gap in the literature.

Similarly, the association between the built environment and youth overweight is often overlooked in research [3]. Of the paucity of research that does exist, it is generally identified that students are less likely to be overweight if their school neighbourhood has access to opportunity structures such as parks, playgrounds, or recreational facilities [3,14]. As such, if we really want to understand child weight status, we must simultaneously examine both individual student characteristics and multiple characteristics of the school environment they attend [13], especially among younger students where the evidence is clearly lacking [15].

As such, the current study seeks to identify potential variability in the odds of a grade 1 to 4 student being overweight and obese across 30 elementary schools, examine the school-level policy and built environment characteristics that explain that variability, and then simultaneously explore which student- and school-level characteristics are associated with overweight and obesity in this sample of young elementary school students.

## Methods

The purpose of the PLAY-On study was to examine the school-level factors associated with PA and overweight among a sample of elementary school students in grades 1 to 8. Given the different data collection protocols required for respondents of this age range, there were two separate study arms of the PLAY-On host study: the first arm of the study was among respondents in grades 5 to 8 (aged 10 to 13 years) and the second arm of the study was among respondents in grades 1 to 4 (aged 6 to 9 years). Research has previously examined the school-level factors associated with overweight [9,10] and PA [11] among the grade 5 to 8 respondents in PLAY-On, and the school-level factors associated with PA among the grade 1 to 4 respondents in PLAY-On [19]. The following study examines the sample of data collected from grade 1 to 4 respondents in PLAY-On. Additional details on the PLAY-On host study are available [9-12].

### Participants

Cross-sectional data were collected in 2007–2008 from a convenience sample of grade 1 to 4 students attending 30 elementary schools in Ontario, Canada. Of the 3,926 students enrolled in grades 1 to 4 at the 30 participating elementary schools, 59.4% (n = 2,331) completed the survey. Missing respondents resulted from parent refusal and absenteeism on the day of the survey. This distribution is consistent with an active consent study examining overweight among Canadian elementary students [17].

### Instruments

As described previously [19], the student-level data were collected from eligible students in grades 1 to 4 using two methods: data provided by parent(s), and data provided by students to the PLAY-On study staff. The school-level data were collected using the two sources. School-level built environment data were provided by the Enhanced Points of Interest (EPOI) data resource. The EPOI data file is a database of the type and location of different opportunity structures within the built environment (recreation facilities variety stores, fast-food restaurants). Additional details about the DMTI-EPOI resources are available online (http://www.dmtispatial.com). The school-level PA policy data were provided by the elementary school version of the Physical Activity Module (PAM) of the Healthy School Planner (HSP). The HSP-PAM is a tool designed to assess policies, activities, committees, facilities and guidelines surrounding PA in the school environment. Additional details about the HSP-PAM measures and assessment categories are available online (http://www.healthyschoolplanner.uwaterloo.ca).

### Procedure

All grade 1 to 4 students at the participating schools were eligible to participate. Prior to participating, active consent from parents (or guardians) was required. Given that parental reports can provide meaningful assessments of PA for children under 12 years of age [20], parents were asked to complete a few questions pertaining to their child’s general PA routines during the active consent process. During the data collection from students, the registered nurse working for PLAY-On asked each eligible student six questions pertaining to their physical activity routines and preferences. After their responses were recorded by the nurse, students were then asked to remove their footwear, and height and weight were objectively measured and recorded by the nurse. Students were asked to stand upright with their back against the wall, and then the nurse used a square placed on the top of their head to measure their height to the nearest centimeter against a standard metric tape measure fastened to the wall. Weight was measured to the nearest one decimal place in kilograms using a Bathscapes® LED digital bath scale (model 1053WWBA). All student data were collected during class time and there was no compensation for participation. At each participating school, the senior administrator(s) most knowledgeable about the school’s policies and resources was asked to complete the HSP survey. The University of Waterloo Office of Research Ethics and appropriate school board ethics committees approved the study procedures.

#### Outcome - weight status

Body Mass Index (BMI) was calculated for each student using the objective measures of weight (kg) and height (m) (BMI = kg/m^2^). Weight status was then determined using the BMI classification system of the International Obesity Task Force [21] based on age and sex adjusted BMI cut-points. Using this system, a student was classified as *overweight* if their age and sex adjusted BMI cut-point was ≥25 and <30, and *obese* if their age and sex adjusted BMI cut-point was ≥30. Students with an age and sex adjusted BMI <25 were classified as normal weight.

#### Student-level measures – parent reports

Given the evidence that parental proxy reports can provide meaningful assessments of PA for children under 12 [22], parents were asked to report “On average, how many hours per day is your child physically active? Please include both moderate (e.g. walking, biking to school, recreational swimming) and vigorous activity.”, and “On an average day, how much time does your child spend watching TV/movies, playing video/computer games, surfing the internet, instant messaging or talking on the phone?”. Based on Canadian PA guidelines for children [23] and consistent with previous research [19], PA for their child was coded as follows: inactive (≤1 hour of PA), moderately active (1 to 3 hours of PA), and very active (>3 hours of PA). Based on Canadian sedentary behaviour guidelines for children [24] and consistent with previous research [19], sedentary behaviour (SB) for their child was coded as follows: low sedentary (≤2 hours of SB), moderately sedentary (2 to 4 hours of SB), and very sedentary (>4 hours of SB).

#### Student-level measures – student reports

Students were asked six questions by the nurse collecting the PLAY-On data. Compared to other kids in your class, when you are at school (during recess, lunch, nutrition breaks) are you…(*more active, less active, about the same*)? Do you prefer to play alone or do you prefer to play with other children (*alone, other children, both*)? Do you prefer active games (e.g. tag, kickball) or do you prefer quiet games (e.g. board games) (*active games, quiet games, both*)? Do you like playing sports (e.g. soccer, basketball) or do you dislike playing sports (*likes sports, dislikes sports*)? Do you like to read or do you dislike reading (*likes reading, dislikes reading*)? Do you like to play outside or do you like to play inside (*outside, inside, both*)?

#### School-level built environment characteristics

There were five different types of opportunity structures examined: recreation facilities (includes dance studios, fitness/gym facilities, and sport and recreation clubs), fast-food restaurants, bakeries/donut shops, variety/convenience stores, and grocery stores (includes supermarkets and mini-markets). Consistent with previous research [9,25], the process of identifying and linking the DMTI-EPOI data to the PLAY-On student level data involved three steps: (1) geocoding the address for each PLAY-On school; (2) creating 1-km circular buffers (i.e., bounded areas surrounding each school in which the six different opportunity structures of the built environment were quantified); and (3) linking the quantified built environment data for each school to the student-level data from each school. Arcview 3.3 [26] software was used to geocode the school addresses and to create the 1-km buffers.

#### School-level physical activity characteristics

As described elsewhere for the PLAY-On study [10,11,19], HSP-PAM measured indicators associated with: Healthy Physical Environment (availability of, access to, and adequacy in meeting student needs for, indoor and outdoor facilities, equipment and resources for safe, quality physical activity on or near school grounds, both during and outside of school hours); Instruction and Programs (availability, delivery and characteristics of curricular physical education, extracurricular physical activity programs, and active transportation to school, including barriers to implementing such programs); Supportive Social Environment (characteristics of the school’s social environment that predispose, reinforce and enable enjoyable, lifelong participation in physical activity or that hinder such activities); and Community Partnerships (the accessibility and availability of support services for physical activity which may include partnerships with public health units and community based services and resources). Each indicator was assigned a classification by the research team based on the corresponding phase of implementation in the Healthy School Continuum as outlined by the Joint Consortium on School Health [27]: *Initiation* (falls short or exhibits extensive room for improvement in meeting the recommendations related to school capacity for physical activity); *Action* (meets the recommendations in several, but not all areas related to school capacity for physical activity, exhibits some room for improvement); *Maintenance* (consistently meets or exceeds the recommendations related to school capacity for physical activity, encouraged to maintain the current level of commitment to supporting physical activity at school). Each of the four main indicators (Healthy Physical Environment, Instruction and Programs, Supportive Social Environment, Community Partnerships) was also assigned an ‘overall’ phase classification based on the combined responses to component indicators. The assessment schemes for the HSP-PAM measures were developed based on the current research literature, Government of Ontario guidelines, and input from experts in the domain of PA in schools. The HSP-PAM does not measure the presence or absence of specific obesity prevention or PA promotion interventions within the school.

### Data analysis

Using student-level data, the prevalence of weight status, parent measures and student measures were examined by sex. Using the school-level data, we calculated the mean and range of each school-level built environment characteristic. Since students (level-1) are nested within schools (level-2), we performed two series of multi-level logistic regression analyses to examine characteristics associated with being (1) overweight versus a normal weight, and (2) obese versus a normal weight. Consistent with other multi-level studies [9,11,12], a three step modelling procedure was used. Step 1 examined if differences in the outcome were random or fixed across schools. The school-level variance term from Step 1 (σ^2^_μ*0*_) was used to calculate the intraclass correlation (ICC) for binary outcomes,^a^ where the ICC represents the proportion of the total variance in student overweight that is due to differences across schools. If significant between-school variability was identified in Step 1, a series of univariate analyses with the school-level characteristics were then performed in Step 2 to identify any school-level characteristics associated with the outcome. In Step 3, a multivariate model was developed to simultaneously examine how the student-level characteristics and the significant school-level characteristics identified in Step 2 were associated with the outcome. Because cross-level interactions between the school-level variables and grade and sex were suspected a priori, these cross-level interactions were also tested while controlling for confounders. If there was no significant between-school variability identified in Step 1, Step 2 was skipped (since there was no school-level variability to be explained by school characteristics), and Step 3 only included the student-level characteristics. After these models were completed, an additional exploratory model was also performed that combined overweight and obese respondents into one category (overweight/obese versus a normal weight). Those data are not shown as significant between-school variability was not identified so it did not provide additional new insight beyond the first two models. The statistical analyses were conducted on ML*wiN* Version 2.02 [28].

## Results

### Student-level weight status

Demographic characteristics of the students are presented in Table 1. The sample was 51.0% (n = 1,187) male and 49.0% (n = 1,139) female. The mean BMI among males was 17.3 (±2.7) kg/m^2^ and 17.1 (±3.0) kg/m^2^ among females. Overall, 13.8% of the sample was considered overweight, 6.2% of the sample was considered obese, and 74.7% were considered normal weight for their age and sex. BMI data were missing from 5.2% (n = 62) of male students and 5.4% (n = 61) of female students. Weight status did not significantly vary by sex (χ^2^ = 0.09, *df* = 3, p = 0.99).

**Table 1:** Descriptive statistics for youth in grades 1 to 4 by sex in PLAY-On (Ontario, Canada, 2007–2008)

		**Male (n = 1,187)**	**Female (n = 1,139)**	**Chi-square**
***Student-level characteristics***		**% (n)**^**a**^	**% (n)**^**a**^	
Weight status^1,b^	Normal weight	75.0 (890)	74.5 (848)	χ^2^ = 0.09, *df* = 3 , p = 0.99
	Overweight	13.7 (162)	14.0 (159)	
	Obese	6.1 (144)	6.2 (71)	
	*Missing*	5.2 (62)	5.4 (61)	
Grade^1^	1	22.7 (270)	22.4 (256)	χ^2^ = 0.32, *df* = 3, p = 0.95
	2	21.5 (255)	21.1 (241)	
	3	27.5 (326)	27.1 (309)	
	4	28.3 (336)	29.4 (335)	
Physical activity level^2^	Low active	10.0 (115)	14.3 (158)	χ^2^ = 11.41, *df* = 2, p < 0.01
	Moderately active	63.3 (726)	62.7 (695)	
	Very active	26.7 (306)	23.0 (255)	
Sedentary behaviour^2^	≤2 hour per day	70.6 (810)	75.2 (834)	χ^2^ = 6.37, *df* = 2, p < 0.05
	2 to 4 hours per day	21.8 (250)	18.9 (210)	
	>4 hours per day	7.6 (87)	5.9 (65)	
Physical activity level compared to other kids^3^	More active	7.4 (86)	9.3 (105)	χ^2^ = 34.56, *df* = 2, p < 0.001
	About the same	28.5 (333)	18.2 (206)	
	Less active	64.2 (750)	72.5 (822)	
Prefers to play alone or with other children^3^	Alone	2.4 (28)	1.0 (11)	χ^2^ = 9.24, *df* = 2, p < 0.01
	Both	57.1 (667)	54.8 (621)	
	Other children	40.5 (474)	44.2 (501)	
Prefers to play active games or quiet games^3^	Active	4.5 (52)	7.6 (86)	χ^2^ = 16.40, *df* = 2, p < 0.001
	Both	41.4 (484)	35.1 (398)	
	Quiet	54.1 (633)	57.3 (649)	
Preference for playing sports^3^	Likes sports	96.5 (1,127)	92.7 (1,049)	χ^2^ = 16.46, *df* = 1, p < 0.001
	Dislikes sports	3.5 (41)	7.3 (83)	
Preference for reading^3^	Likes reading	79.6 (931)	89.5 (1,014)	χ^2^ = 42.66, *df* = 1, p < 0.001
	Dislikes reading	20.4 (238)	10.5 (119)	
Prefers to play outside or inside^3^	Outside	4.1 (48)	4.2 (48)	χ^2^ = 4.35, *df* = 2, p = 0.11
	Both	20.6 (241)	17.2 (195)	
	Inside	75.3 (880)	78.6 (890)	

^a^Numbers may not add to total because of missing values.

^b^Body mass index (BMI) values used to determine weight status have been adjusted for age and sex.

^1^Measured by PLAY-On nurse.

^2^Reported by parent or guardian.

^3^Reported by student and recorded by PLAY-On nurse.

PLAY-On represents the name of the study where self-reported data were collected in 2007–2008 from a convenience sample of students in grades 1 to 4 attending 30 elementary schools in Ontario, Canada.

### School-level characteristics

The mean number of fast food retailers within a 1-km buffer of the schools was 1.7 (range, 0 to 8). The mean number of bakeries/donut shops within a 1-km buffer of the schools was 1.1 (range, 0 to 8). The mean number of variety stores within a 1-km buffer of the schools was 1.3 (range, 0 to 8). The mean number of grocery stores within a 1-km buffer of the schools was 1.8 (range, 0 to 13). The mean number of recreation facilities within a 1-km buffer of the schools was 0.8 (range, 0 to 4). The majority of schools were in the action phase for the overall indicator scores for Healthy Physical Environment (66.7%) and Supportive Social Environment (66.7%) and the maintenance phase for the overall score for Community Partnerships (56.6%). Conversely, the majority of schools were in the initiation phase for the overall score for Instruction and Programs (73.3%). None of the schools were in the maintenance phase for the overall scores for Healthy Physical Environment, Instruction and Programs, and Supportive Social Environment. Within each of the main indicator categories, there was substantial variability across schools in relation to the individual indicators measured within each category. The descriptive statistics for HSP-PAM indicators for the PLAY-On study are available elsewhere [11].

### School characteristics associated with overweight

Among students in grades 1 to 4, significant between-school random variation in the odds of being overweight was identified [σ^2^_μ0_ = 0.274(0.106), p < 0.001]; school-level differences accounted for 7.7% of the variability in the odds of a student being overweight versus a normal weight. As shown in Table 2, the univariate analyses revealed that two school-level PA policy characteristics were significantly associated with the likelihood of a student being overweight; none of the built environment characteristics surrounding schools were significantly associated with the likelihood of a student being overweight.

**Table 2:** Results of the univariate multi-level logistic regression analyses examining school-level physical activity policy characteristics associated with being overweight among youth in grades 1 to 4 in PLAY-On (Ontario, Canada, 2007–2008)

	**Model estimates (standard error)**	
		**Model 1**
		**Overweight vs. normal weight **^**a**^
	**Phase**	
**Healthy physical environment**		
Student access to a variety of facilities on and off school grounds during school hours^**†**^	Action	1.64 (0.71)*
	Maintenance	1.15 (0.71)
Availability of physical activities during inclement weather^**†**^	Action	0.45 (0.29)
	Maintenance	−0.18 (0.95)
Student access to facilities and equipment outside of school hours^**†**^	Action	−0.22 (0.35)
	Maintenance	0.77 (0.69)
Support for active transportation to and from school^**†**^	Action	−0.43 (0.37)
	Maintenance	−0.84 (0.39)*
***Overall score for this indicator***^**†**^	*Action*	−0.14 (0.28)
**Instruction and programs**		
Implementation of daily PA^‡^	Maintenance	−0.23 (0.39)
Time spent per week engaged in PA during physical education classes^**†**^	Action	−0.23 (0.44)
	Maintenance	−0.18 (0.58)
Classes taught by a qualified physical education specialist^**†**^	Action	−0.12 (0.32)
Availability and use of intramural/club activities^**†**^	Action	−0.06 (0.26)
	Maintenance	−0.10 (0.29)
Consistency of intramural programming across grade divisions and seasons^**†**^	Action	0.09 (0.46)
	Maintenance	0.36 (0.95)
Availability and use of interschool programs^**†**^	Action	−0.02 (0.35)
	Maintenance	−0.33 (0.54)
Consistency of interschool programming across seasons^**†**^	Maintenance	−0.13 (0.37)
***Overall score for this indicator***^**†**^	*Action*	0.15 (0.30)
**Supportive social environment**		
Emphasis placed on maximizing participation in PA through school programs^**†**^	Action	1.03 (0.70)
	Maintenance	1.08 (0.65)
Incorporation of PA into other school subjects^**†**^	Action	0.32 (0.54)
	Maintenance	0.56 (0.64)
Special recognition of students who participate in school physical activities^**†**^	Action	0.74 (0.63)
	Maintenance	−0.09 (0.40)
Formal collection of suggestions from the school community about PA at school^**†**^	Action	0.02 (0.66)
	Maintenance	0.06 (0.44)
Promotion of PA programs and events for students, families and school staff^**†**^	Action	−0.81 (0.59)
	Maintenance	−0.43 (0.47)
Use of PA as a reward, not as discipline^**†**^	Action	−0.12 (0.48)
	Maintenance	−0.24 (0.53)
Presence of written policies or practices that support PA^**†**^	Action	−0.14 (0.28)
	Maintenance	−0.06 (0.22)
***Overall score for this indicator***^**†**^	*Action*	−0.37 (0.20)
**Community Partnerships**		
Support available for school staff involved with PA^‡^	Maintenance	−0.50 (0.30)
Connection to community resources^**†**^	Action	−0.40 (0.42)
	Maintenance	−0.19 (0.35)
***Overall score for this indicator***^**†**^	*Action*	−0.56 (0.29)
	*Maintenance*	−0.05 (0.17)
**Number of opportunity structures within a 1 kilometer (km) radius of a school**		
Recreation facilities	*Each 1 unit increase*	−0.02 (0.09)
Variety stores		−0.13 (0.11)
Fast-food restaurants		−0.19 (0.14)
Bakeries/donut shops		0.05 (0.13)
Grocery stores		−0.13 (0.08)

Note: ^a^BMI values used to determine weight status have been adjusted for age and sex.

Model 1: 1 = Overweight (n = 319), 0 = Normal weight (n = 1,711).

*p < .05 ^†^Reference group is Initiation, ^‡^Reference group is Action.

### Significant school- and student-level characteristics associated with overweight

The adjusted odds ratios for the significant school and student characteristics associated with overweight are presented in Table 3 (Model 1). Students considered very active (OR 0.53, 95% CI 0.35 to 0.81) or moderately active (OR 0.65, 95% CI 0.45 to 94) were less likely to be overweight compared to students considered low active. Students who reported they prefer to play active games were less likely to be overweight than students who reported they prefer to play quiet games (OR 0.54, 95% CI 0.30 to 0.97). Conversely, students spending 2 to 4 hours per day in sedentary behaviours were more likely to be overweight compared to students spending less than 2 hours per day in sedentary behaviours (OR 1.55, 95% CI 1.18 to 2.04). There were two school characteristic associated with being overweight. First, if a student attended a school that was in the action or maintenance phase for the indicator *Student access to a variety of facilities on and off school grounds during school hours*, he/she was less likely to be overweight than a similar student attending a school that was in the initiation phase for this indicator (OR 0.39, 95% CI 0.16 to 0.92 and OR 0.32, 95% CI 0.12 to 0.86 respectively). Second, if a student attended a school that was in the maintenance phase for the indicator *Support for active transportation to and from school*, he/she was less likely to be overweight than a similar student attending a school that was in the initiation phase for this indicator (OR 0.47, 95% CI 0.22 to 0.98). These two school-level characteristics explained 7.3% of the between school variability in overweight. There were no significant contextual interactions identified between these two school-level characteristics and sex or grade pertaining to the odds of being overweight.

**Table 3:** Multi-level logistic regression analyses of school- and student-level characteristics associated with overweight and obesity among youth in grades 1 to 4 in PLAY-On (Ontario, Canada, 2007–2008)

***Student-Level dharacteristics***		**Adjusted odds ratio**^**§ **^**(95% CI)**	
		**Model 1**	**Model 2**
		**Overweight vs. normal weight**^**a**^	**Obese vs. normal weight**^**a**^
Sex	Female	1.00	1.00
	Male	0.90 (0.70, 1.17)	1.01 (0.71, 1.44)
Grade	1	1.00	1.00
	2	0.68 (0.46, 1.01)	1.47 (0.88, 2.44)
	3	0.80 (0.56, 1.14)	1.41 (0.85, 2.34)
	4	1.07 (0.76, 1.51)	0.98 (0.57, 1.69)
Physical activity level	Low active	1.00	1.00
	Moderately active	0.65 (0.45, 0.94)*	0.75 (0.46, 1.20)
	Very active	0.53 (0.35, 0.81)**	0.50 (0.28, 0.89)*
Sedentary behaviour	≤2 hour per day	1.00	1.00
	2 to 4 hours per day	1.55 (1.18, 2.04)**	1.49 (1.01, 2.19)*
	>4 hours per day	1.64 (0.53, 5.11)	1.62 (0.36, 7.22)
Physical activity level compared to other kids	About the same	1.00	1.00
	More active	0.79 (0.46, 1.37)	1.26 (0.60, 2.62)
	Less active	0.95 (0.70, 1.28)	1.30 (0.82, 2.06)
Prefers to play alone or with other children	Alone	1.00	1.00
	Other children	0.57 (0.25, 1.34)	0.90 (0.26, 3.09)
	Both	0.48 (0.20, 1.11)	0.49 (0.14, 1.71)
Prefers to play active games or quiet games	Quiet	1.00	1.00
	Active	0.54 (0.30, 0.97)*	0.76 (0.35, 1.64)
	Both	0.96 (0.73, 1.25)	0.84 (0.58, 1.24)
Preference for reading	Dislikes reading	1.00	1.00
	Likes reading	0.96 (0.68, 1.35)	0.98 (0.60, 1.60)
Prefers to play outside or inside	Inside	1.00	1.00
	Outside	1.03 (0.56, 1.92)	0.87 (0.36, 2.07)
	Both	1.45 (0.99, 2.05)	0.92 (0.57, 1.50)
***School-Level Characteristics***			
**Healthy Physical Environment**			
Student access to a variety of facilities on and off school grounds during school hours	Initiation	1.00	-
	Action	0.39 (0.16, 0.92)**	
	Maintenance	0.32 (0.12, 0.86)**	
Support for active transportation to/from school	Initiation	1.00	-
	Action	0.72 (0.36, 1.42)	
	Maintenance	0.47 (0.22, 0.98)*	

Note: § Odds ratios adjusted for all other variables in the table and controlling for random variation across schools.

^a^BMI values used to determine weight status have been adjusted for age and sex.

Model 1: 1 = Overweight (n = 319), 0 = Normal weight (n = 1,711).

Model 2: 1 = Obese (n = 143), 0 = Normal weight (n = 1,711).

*p < .05 **p < .0.

PLAY-On represents the name of the study where self-reported data were collected in 2007–2008 from a convenience sample of students in grades 1 to 4 attending 30 elementary schools in Ontario, Canada.

### School-level characteristics associated with obesity

No significant between-school random variation in the odds of being obese was identified [σ^2^_μ0_ = 0.115(0.089)]. This suggests that among students in grades 1 to 4, school-level differences did not account for a significant amount of the variability in the odds of a student being obese versus a normal weight. As such, there was no need to examine potential associations between the school level characteristics and obesity.

### Significant student-level characteristics associated with obesity

The adjusted odds ratios for the student characteristics associated with obesity are presented in Table 3 (Model 2). Students considered very active were less likely to be obese compared to students considered low active (OR 0.50, 95% CI 0.28 to 0.89). Conversely, students spending 2 to 4 hours per day in sedentary behaviours were more likely to be obese compared to students spending less than 2 hours per day in sedentary behaviours (OR 1.49, 95% CI 1.01 to 2.19).

## Discussion

Using objectively measured height and weight, we identified significant differences in the likelihood of students in grades 1 to 4 being overweight across schools; we did not find significant between-school variability in the likelihood of being obese. The finding that individual student overweight varies as a function of the school a student attends is consistent with similar research on students in higher grades in elementary school [9,14,17], and secondary schools [2]. Considering that a recent systematic review identified that interventions to prevent overweight are ineffective among preschool children [29], our results suggest that there is the potential for overweight and obesity prevention programs to be effective if robust interventions are implemented early in elementary school settings; this would require evaluation. It should be noted that primary prevention efforts should focus on the large population of students who are not yet overweight, and any secondary prevention efforts should be targeted at the school contexts that increase the risk of overweight and the sub-populations of youth considered high risk.

The importance of creating healthy school environments through school policies was supported with this research. First, students in grades 1 to 4 were less likely to be overweight if they attended a school that allowed student access to recreational facilities on and off school grounds during school hours. This is consistent with a recent Institute of Medicine report [30] which recommends local governments work with schools to institute policies to mandate minimum levels of play space, equipment for physical activity, and duration of play in schools. Our finding is also consistent with research among older students in the PLAY-On study showing that grade 5 to 8 students are more active if they attend a school that has a policy to provide students with access to facilities and equipment outside of school hours [11]. Considering that (a) activity can be incorporated in the school curriculum without negative academic consequences (while also providing physical, social and emotions benefits for students) [31], (b) research suggests that in school activity programming can make a substantial contribution to the activity levels of kids (especially in rural settings or among youth from low socioeconomic contexts) [32], and (c) recess is a core part of the curriculum in elementary school due to the impact it has on increasing student activity levels [33], it may be prudent for elementary schools to maintain or implement policies that ensure students have access to a variety of facilities and resources to support PA during the school day as a mechanism to promote healthy weights among students. However, considering research has shown that among youth of this age group, simply providing children with access to facilities and equipment during the school day (i.e., at recess) does not increase time spent being active [33], policies providing students with access to facilities at school may also need to be supplemented with programming designed to provide additional supervision, guidance and encouragement from school staff.

Second, students in grades 1 to 4 were less likely to be overweight if they attended a school that strongly supported active transport to and from school. This is consistent with research that suggests active transportation to and from school is associated with lower BMI among youth [34-36]. Research suggests that active transport programs should be implemented in the first year of elementary school and maintained as children progress through school because such programs are associated with favourably influencing the trajectory of youth BMI [35]. This finding was also consistent with the Institute of Medicine [30] which recommends local governments work with communities and schools to implement a Safe Routes to School program that increases the number of students who can actively commute to schools. Considering that research suggests that each extra kilometer a child walks per day reduces their likelihood of being obese by 4.8% [37], if schools could provide additional supervision to allow young students to have safe routes for actively commuting before and after the school day (or walking a short distance to get to and from the school bus in rural schools) the potential population-level impact across all students in elementary schools over the school year could be substantial.

The current study did not find a significant association between characteristics of the built environment surrounding a school and overweight or obesity among grade 1 to 4 students. This is in contrast to some previous work among older children and youth [3,14,38], but consistent with both previous research with grade 5 to 8 students in PLAY-On [9] and evidence examined in a recent systematic review [15]. Considering that research has suggested that the built environment may not have as large of an impact on overweight among younger students [6,39], it may be more important for future research among elementary school-aged youth to consider the built environment in the neighbourhood where the child lives rather than at their school for this age group.

Despite research suggesting that children’s activity preferences are an important determinant of their PA behaviour [7], this study identified that children’s activity preferences were not generally associated with overweight or obesity. The only preference identified that was associated with a lower likelihood of being overweight was children who reported that they prefer active games, a finding consistent with research highlighting that children who prefer quiet games and activities are less physically active than children who prefer active games [40]. Although not identified in this study as being important predictors of overweight and obesity, given the “gate-keeper” role that parents and schools often play in providing activity opportunities for children [41], understanding the activity preferences of students may help to inform the types of interventions provided in the school context.

### Limitations

Data were not available to examine energy intake in the present study so our understanding of student-level characteristics associated with obesity is limited to aspects of the energy expenditure side of the caloric balance equation. Furthermore, data pertaining to food policies within a school (e.g., vending machines, cafeteria options) were not available; factors which may explain some of the between-school variability in overweight identified. It was also not possible to examine the impact of specific school-based programs and interventions which may have been in place in the participating schools and their potential interactions with opportunity structures in the built environment. Causal relationships cannot be inferred from these cross-sectional data.

## Conclusions

It is unlikely that current trends in childhood overweight and obesity can be reversed without more effective school-based obesity prevention programming. Research recommends targeting obesity prevention efforts at the modifiable characteristics of school environments that put students at risk because of the significant amount of time youth spend in the school environment. The results from the present study suggest that it may be wise to target obesity prevention efforts to schools that do not provide student access to recreation facilities during school hours or schools that do not support active transportation for students. It is important to understand the school characteristics that may influence behaviour because if a school program or policy can be changed to cause even a small impact either shifting or normalizing the distribution of a risk factor across all schools, the effect across all students could be substantial. Future research should evaluate if the optimal population level impact for school-based overweight and obesity prevention programming might be achieved most economically if interventions selectively targeted the schools that are putting students at the greatest risk (i.e., schools that do not provide student access to recreation facilities during school hours or schools that do not support active transportation for students) and that such programs need to start early in elementary school settings.

### Human subjects approval statement

The University of Waterloo Office of Research Ethics and appropriate school board ethics committees approved the study procedures.

## Endnote

^a^The formula for calculating the intraclass correlation (ICC) for binary outcomes, where ICC=σ2μ0σ2μ0+π23.

## Competing interest

The author declares that he has no competing of interest.

## Pre-publication history

The pre-publication history for this paper can be accessed here:

http://www.biomedcentral.com/1471-2458/13/982/prepub
